# Being useful among persons aged over 65: social representations from a cross-sectional European study

**DOI:** 10.1007/s40520-020-01767-x

**Published:** 2021-01-03

**Authors:** Paul de Boissieu, Serge Guerin, Véronique Suissa, Fiona Ecarnot, Aude Letty, Stéphane Sanchez

**Affiliations:** 1 Department of Epidemiology and Public Health, Kremlin-Bicêtre University Hospital APHP, Le Kremlin-Bicêtre, France; 2 Korian, (The Korian Foundation for Ageing Well), Paris, France; 3 Laboratoire de Psychopathologie et Neuropsychologie EA2027, Université Paris VIII, Paris, France; 4 EA3920, University of Burgundy Franche-Comté, 25000 Besancon, France; 5 Department of Cardiology, University Hospital Besançon, Besançon, France; 6 Centre Hospitalier de Troyes, (Pôle Territorial Santé Publique & Performance), Troyes, France; 7 EA3797-Viellissement Fragilité, URCA, Reims, France

**Keywords:** Ageing, Useful, Ageing-well, Europe

## Abstract

**Background:**

There is a compelling need to prepare our societies and healthcare systems to deal with the oncoming wave of population ageing. The majority of older persons maintain a desire to be valued and useful members of society and of their social networks.

**Aims:**

We sought to investigate the perception of usefulness among persons aged 65 years and over in four European countries.

**Methods:**

We performed a cross-sectional survey with a representative sample of individuals aged 65 years or older from the population of retired persons (including recently retired persons and oldest-old individuals) from 4 European countries selected using quota sampling. In February 2016, an internet questionnaire was sent to all selected individuals. The characteristics used for the quota sampling method were sex, age, socio-professional category, region, city size, number of persons in household, autonomy, marital status, place of residence, income and educational status. The questionnaire contained 57 questions. Sociodemographic characteristics were recorded. Responses were analysed with principal components analysis (PCA).

**Results:**

A total of 4025 persons participated; 51% were males, and 70% were aged 65–75 years. PCA identified six classes of individuals, of which two classes (Classes 2 and 3) were characterized by more socially isolated individuals with little or no sense of usefulness, low self-esteem and a poor sense of well-being. These two classes accounted for almost 20% of the population. Younger and more autonomous classes reported a more salient sense of usefulness.

**Conclusions:**

The loss of the sense of usefulness is associated with dissatisfaction with life and a loss of pleasure, and persons with profiles corresponding to Classes 2 and 3 should, therefore, be targeted for interventions aimed at restoring social links.

**Supplementary Information:**

The online version contains supplementary material available at 10.1007/s40520-020-01767-x.

## Introduction

The population of the world is ageing, with the exception of Africa [1]. Europe is particularly affected by population ageing, a phenomenon that is likely to become more marked in the coming decades. In 2018, 19% of the inhabitants of the European Union (EU) were aged 65 years or over, and this proportion is set to increase to 28.5% by 2050 [2]. The proportion of persons aged 80 years and over is on track to double within the coming 30 years, from 5.5% in 2018, to 11% by 2050 [3]. There is, therefore, a compelling need to prepare our societies and healthcare systems to deal with this oncoming wave of ageing.

For the individual, ageing, and particularly passing the landmark 65 years of age, is characterized by progressive changes in one’s lifestyle, social links and health status. Advancing age can also be marked by structural life changes, which may be experienced in a positive or negative light. Retirement, for example, can be seen as an opportunity, or alternatively, as a transition that is difficult, more akin to being socially demoted. For some people, retirement is a welcome rest after many years of hard work. In this paradigm, it is seen as a new life phase with more leisure-time opportunities for personal development. Conversely, other people may see retirement as a form of exclusion from society, or social isolation, synonymous with boredom [4]. Unhappy life events (such as illness or bereavement) can also change a person’s outlook on their ageing [5]. Despite these different possible perspectives, it would appear that the majority of older persons maintain a desire to be valued and useful members of society and of their social networks [6]. Indeed, a sense of purpose and being of help to others was found to be one of the main components of successful ageing among grandparents [7]. This feeling of usefulness has also been identified as a strong predictor of health status (notably disability and mortality) in older persons [6] and is considered to be one of the major challenges of ageing, particularly successful ageing [8].

The feeling of usefulness is subjective to a large degree, and the perception can vary widely from one person to the next [9]. Among the factors that can negatively impact one’s feeling of usefulness, societal stereotypes about older persons play a key role. Ageing is often seen as a burden on society [10], and growing old is thought to be synonymous with dependency, illness, poverty and isolation [11]. These stereotypes of ageing are prejudicial to the perception that people have of their own ageing process [12]. In addition, this representation of generational opposition (pitting the young and useful against the old and non-productive) could convey a false perception of the utility of older subjects, thereby constituting a factor that could modulate successful ageing.

Yet, although each person can have their own ideas about what it means to be useful, the concept remains largely determined by the social and cultural context (e.g. family origins, beliefs, geographic location, family situation, life course….), as well as by financial and health status in older age [13].

There is a paucity of data on the perception of usefulness among older subjects [14–16]. The few available studies indicate that older adults with persistently low feelings of usefulness, or whose feeling of usefulness is declining, are at greater risk of death (adjusted hazard ratio = 1.75; 95% confidence interval = 1.22, 2.51) [14]. Knowing what it means to older persons to be useful could help to orient suitable interventions on this problematic societal question. It would first help to identify persons who have a low estimation of their own usefulness. Indeed, a Chinese study of 29,954 observations from 19,070 older adults aged 65 and older found that persistent self-perceived uselessness was associated with negative ageing outcomes in terms of autonomy, cognitive function and life satisfaction [17]. Second, it would be helpful to identify subgroups of older persons who share a similar degree of feeling of usefulness (or lack thereof). Indeed, taking into account the factors associated with this poor perception of usefulness could guide the design and implementation of targeted interventions to boost the feeling of usefulness among those who are most in need.

The aim of this study was, therefore, to evaluate the perception of usefulness among persons aged 65 years and over in four European countries.

## Methods

### Study population

This survey was carried out in February 2016 by a polling institute in a representative sample of individuals from the population of retired persons in four European countries, selected using quota sampling. Quota sampling is a sampling method that consists in ensuring that the sample is representative of the overall population, by adopting a similar structure to the overall population from which the sample is taken, in terms of several criteria, such as age distribution, socio-economic status, etc.

A total of 4025 subjects aged 65 years or older were selected. This cross-sectional study was carried out using an internet questionnaire sent to the selected individuals in France (n = 1000), Belgium (n = 1011), Italy (n = 1012) and Germany (n = 1002). The survey was based on quota sampling to achieve a sample representative of the general population. The reference population selected was diverse in terms of age, including both recently retired persons as well as oldest-old individuals. The characteristics used for the quota sampling method were sex, age, socio-professional category, region, city size, number of persons in the household, autonomy (dependent/independent), marital status, place of residence, income and highest educational qualification. The quotas were defined based on the socio-demographic structure of the population. The aims of the survey were also to obtain information about the different subgroups of the population (according to age, sex, income, etc.).

### The Ipsos© Access Panel

The questionnaire contained 57 questions and was administered via the Access Panel online service belonging to Ipsos Interactive Services^**©**^. The Access Panel is a pool of households and individuals spread homogeneously across the whole of each country and who regularly accept to participate in market research studies. The panel comprises over 600,000 individuals on whom detailed information has been collected in addition to the data used to establish the quotas (e.g. the size of the household, income, level of education, number of children etc.). This methodology guarantees satisfactory representativeness for all quotas (i.e. sex, age, socio-professional category, city size and region). Numerous quality controls were carried out at all stages of the survey. Participants received a small financial incentive for their participation.

### Quality control procedures

Quality control procedures were implemented at each stage of data collection and all the online sessions were performed using the CONFIRMIT software (Confirmit AS, Oslo, Norway). This system enables automatic management of how the questionnaire scrolls (guides, filters), eliminates coding errors (e.g. the system does not allow two answers to be given for a question where only one answer is required), displays questions or sub-questions in random order to avoid bias linked to the order of appearance of the items on the questionnaire, controls coherence between responses and ensures automated control of quotas in real time. This software meets ISO 9001 certification standards (2008 version).

### Development of the questionnaire

The questionnaire used for this study was developed jointly by the Korian Foundation for Ageing Well, IPSOS^**©**^, and a sociologist. The questionnaire contained 57 questions and was designed specifically for this study by the investigators. The questionnaire was not pilot tested or validated prior to this study. The English translation of the questionnaire is provided in the Supplementary Material.

### Statistical analysis

For descriptive analysis, qualitative variables are presented as number and percentage. Data were analysed using Principal Components Analysis (PCA). PCA is a method for reducing the dimensionality of very large data sets, by transforming a large number of variables into a smaller set that nonetheless retains most of the information. In brief, the process of PCA consists in computing “principal components”, i.e. new variables that are linear combinations of the initial variables and that contain a maximum of information from the initial, large dataset. The principal components are uncorrelated between themselves (so as to represent distinct and independent concepts or profiles), were performed based on questions 38, 41, 43, 44 and 57 as active variables, while questions 1 to 37, 39, 42, 49 and 56 were used as illustrative variables. Active variables are used to compute the principal components. Illustrative variables have no impact on the construction of the principal components, but rather are only used to help with interpretation.

## Results

In total, 4025 subjects were included in this analysis. The main characteristics of the study population are displayed in Table 1 (overall, and by class resulting from the PCA): 51% were males, 49% were females, 70% were aged 65–75 years and 409 (10%) were aged over 80. A total of 2715 (67%) were married or living maritally, while 3083 subjects (75%) had at least one other person living in their household. The level of education was low (primary school and/or some secondary school) in 919 subjects (23%), and 950 subjects (28%, 718 missing data) declared that they had a low level of income (< 1700 Euro per month).

**Table 1:** Characteristics of the Study Population

Characteristic	Total (*N* = 4025): 100%	Class 1 (*N* = 577):14% (%)	Class 2 (*N* = 195): 5% (%)	Class 3 (*N* = 593):15% (%)	Class 4 (*N* = 959): 24% (%)	Class 5 (*N* = 758): 19% (%)	Class 6 (*N* = 933):23% (%)
**Country**							
Germany	**24.9**	10.8	8.2	30.9	21.1	31.6	31.6
Belgium	**25.1**	16.3	10.3	26.6	23.4	38.6	23.4
France	**24.8**	25.5	24.4	19.8	37.3	11.6	25.9
Italy	**25.1**	47.4	57.1	22.7	18.1	18.2	19.2
**Sex**							
Men	**42.6**	41.8	41.2	47.6	45.0	44.4	36.1
Women	**57.4**	58.2	58.8	52.4	55.0	55.6	63.9
**Age (years)**							
65–69	**28.5**	26.1	21.4	24.7	27.0	30.6	33.5
70–74	**22.5**	23.0	15.1	19.7	21.9	23.5	25.2
75–79	**29.4**	29.2	31.7	32.2	29.6	31.1	25.8
≥ 80	**19.6**	21.7	31.8	23.4	21.6	14.8	15.5
**Household**							
Living alone	**27.9**	16.9	17.9	34.3	29.9	29.1	29.7
≥ 2 members	**72.1**	83.1	82.1	65.7	70.1	70.9	70.3
**Marital status**							
Single	**37.3**	30.1	41.5	47.9	36.9	35.5	36.2
Couple	**62.7**	69.9	58.5	52.1	63.1	64.5	63.8
**Grandchildren**							
None	**26.6**	25.3	28.4	35.9	22.9	28.9	23.0
≥ 1	**73.4**	74.7	71.6	64.1	77.1	71.1	77.0
**Acts as carer**							
Yes	**64.0**	67.1	50.2	50.2	60.7	66.7	75.0
No	**36.0**	32.9	49.8	49.8	39.3	33.3	25.0
**Level of education**							
Low (primary/some secondary)	**26.5**	30.6	46.6	33.2	22.8	22.1	23.1
Medium (secondary and ≤ 2 years higher level)	**43.6**	49.3	41.1	40.6	44.0	43.3	42.5
High (university degree or higher)	**29.8**	20.2	12.4	26.1	33.3	34.6	34.4
**Monthly Income**							
Low (< 1700 Euro)	**25.7**	21.9	35.7	35.3	22.9	26.8	21.6
Medium (1700–2999 Euro)	**31.1**	32.1	29.0	27.0	31.7	32.8	31.5
High (≥ 3000 Euro)	24.6	25.7	18.3	19.2	27.8	22.8	26.8

Principal components analysis (PCA) was performed using questions 38, 41, 43, 44 and 57 as active variables, while questions 1 to 37, 39, 42, 49 and 56 as illustrative variables. PCA identified six distinct classes (Table 2). The main characteristics of the six classes are presented in Table 1.

**Table 2:** Characteristics of the 6 classes identified by principal components analysis

	TOTAL *N* = 4025 (100%) (%)	Class 1 N = 577 (14%) (%)	Class 2 N = 195 (5%) (%)	Class 3 *N* = 593 (15%) (%)	Class 4 *N* = 959 (24%) (%)	Class 5 *N* = 768 (19%) (%)	Class 6 *N* = 933 (23%) (%)
Feeling of utility	80.0	82.4	56.4	60.0	76.3	89.0	92.7
Satisfied with their life	83.1	83.8	66.6	71.3	80.2	88.4	92.0
Life is a source of pleasure	75.1	72.9	45.9	61.6	71.9	82.1	88.6
Aging well	74.4	73.6	60.9	64.0	71.1	81.6	81.8
Feels lonely	51.3	58.0	72.7	60.6	51.8	45.0	41.6
Suffers from loneliness	23.5	26.3	41.1	32.5	24.2	19.4	15.1
Has no-one to help out if feeling down or unwell	8.3	6.4	11.7	17.5	8.2	6.3	4.8
Has a family member in a nursing home If yes, visits family member	14.3 83.2	16.2 88.3	11.2 77.7	12.0 76.7	14.9 75.8	13.7 84.5	15.2 89.5

Class 1 (*N* = 549, 14%): Comprised mainly of Italians, this class is older than the average of the other classes, with more females (Table 1). They share a feeling that older people do not receive adequate recognition in society. They are surprised by the idea that an older person could take up studies, refuse to take retirement once they have reached the official retirement age, or start a new life for themselves. This group considers that their utility in life evolves over time and is centred around their family and the help they can provide to their nearest and dearest. They know what to do to make themselves useful to others, but this activity does not give them a strong sense of accomplishment. Indeed, they have a higher than average tendency to report a low level of physical and mental well-being and have low self-esteem.

Class 2 (*N* = 169, 5%): Again comprised mainly of Italians, the class is almost on the margins of society. Similar to Class 1, the members of this group are generally older than average, and more often female (Table 1). They consider that older people should not have any significant role in society. They are also incensed by changes in lifestyle in elderly persons (e.g. taking up studies, re-marrying….). They are distinct from Class 1 in that they feel less useful. Little or nothing makes them feel as though they are useful. Life is no longer a source of pleasure for them, and they feel less confident than average about the future. The individuals in this class are lonelier than average and are often widowed.

Class 3 (*N* = 563, 15%): Comprising a majority of German individuals, this class has more single people than average and a high representation of people aged 80 years and over. The level of education and income in this class is generally low (Table 1). They declared less frequently than average that they can rely on a strong family core and are often dependent on assistance from other people. The defining feature of this Class is that no “useful” activity is considered likely to procure them a feeling of accomplishment. They seem to be more isolated than those in Class 2 and more often declare that they never have any opportunity to be useful to others. They are characterised by a strong sense of being useless. They make no plans for the future and do not find life to be a source of pleasure.

Class 4 (*N* = 965, 24%): Composed in large part of French people, the members of this Class have an intermediate level of education and income (Table 1). They are surrounded by their families and consider more often than average that the feeling of utility is built around their own autonomy and the help they can provide to others (volunteering, community service…). Their independence is key, and the help they provide to others contributes less to their feeling of utility than the activities they do for and by themselves. This attachment to their independence makes them hostile to anything that might curb it. They are generally unsatisfied with their current life and do not have confidence in the future.

Class 5 (*N* = 799, 19%): Belgians and Germans are predominant among the members of this Class. They are younger and more physically fit than average (Table 1). They more frequently declare that they are in full possession of their faculties and can do everything alone. They are characterised by a higher level of well-being than the previous four classes. They are very satisfied with their lives. They are optimistic and are distinct from the other classes in that they more often think that it is possible to help others even after losing one’s autonomy and even when one lives in a nursing home. They have a strong sense of being useful and more often consider that it is possible to feel useful when living in a nursing home. They are particularly hostile to any measure that might limit their life choices. The present is a source of pleasure for them and they have confidence in the future.

Class 6 (*N* = 980, 23%): Composed of a majority of Germans, this class has a younger average age and a higher than average proportion of females (Table 1). As in class 5, the members of this class have a strong feeling of accomplishment, are in good physical health and have a good network of social support. Most of their personal daily tasks procure them a strong feeling of utility, but they also feel useful through the help they provide to others. Any “action” in their daily life contributes to this feeling of utility, such as things they do for themselves, for their family, for others, or activities for the community or associations. This participation is more important for this class than for the others. The members of class 6 consider that nothing should stifle their investment in making themselves useful. This strong attachment to their independence is mirrored by a very strong desire to keep their autonomy when living in a nursing home. Indeed, they feel it is essential for nursing home residents to be able to choose their own clothes, store their own belongings, do their own shopping, etc.

## Discussion

In this study, we found that more than 80% of the 4025 participants aged 65 and over had a feeling of being useful. However, there is wide variability, explored with PCA, which described six distinct classes of subjects.

Two of the Classes (2 and 3) accounted for almost 20% of the population and were characterised by a less salient feeling of utility (55–60%) than the rest of the study population. Despite these similar characteristics, subjects in Class 2 were older, had a more negative view of their ageing process than those in Class 3 and might be considered as experiencing “unsuccessful” ageing. Both Class 2 and Class 3 have an older population that is also more socially isolated than the rest of the population. Loneliness and the breakdown of social links are already known to be risk factors for the occurrence of negative health outcomes [18, 19].

Our study brings to light new aspects associated with the loss of social links. The weakening of social contact seems to be associated with a reduction in the feeling of utility. Social interactions among elders generally tend to decrease due to such factors as being widowed, having children and grandchildren who live far away, seeing ones’ contemporaries die, or because of reduced resources. Getting involved socially can also be affected by health problems or loss of autonomy, resulting in significantly reduced social participation. The person subsequently risks feeling that they are no longer masters of their own choices, further eroding their feeling of utility. The loss of the sense of usefulness is associated with dissatisfaction with life and a loss of pleasure, and the subjects in Classes 2 and 3 should, therefore, be targeted for interventions that aim to restore social links. Indeed, in the fight against loneliness, it has been shown that the most socially isolated individuals were those most in need of such initiatives [20].

A range of different types and formats of intervention can be envisaged to help restore the feeling of utility among older individuals. Actions may be of two types, namely individual or collective. Collective interventions seem to achieve the best results and should be preferred over individual interventions [21, 22]. Collective interventions should not simply consist in bringing a group of lonely elders together in a meeting or workshop. Indeed, because of their loneliness and associated social behaviours, simply bringing them together in a group situation is not likely to be sufficient to create a social link between them [23]. Various activities have been proposed to combat loneliness among older people (e.g. social or physical activity, discussion and support groups, internet use, home visits…). The benefits of these activities are varied and include reduced loneliness, increased social support, improved physical and mental health. However, no single intervention seems capable of achieving an improvement across all these domains [21]. Accordingly, no single intervention appears to be better than the others, thus precluding recommendation of any one specific type of intervention aimed at boosting the feeling of utility among older individuals. To maximise the benefits, programmes combining several different types of interventions and targeting different domains could be proposed [24].

Different cultures form different views and norms about the role older people should play in society and also about how older persons are treated within society. Cultures with strong family values, where it is still common for multi-generation families to live under the same roof may provide more opportunities for older persons to engage and feel useful. A recent meta-analysis comprising samples from 23 countries totalling 21,093 total participants directly compared Eastern and Western cultures in their attitudes toward ageing and the aged and found that contrary to popular belief, Eastern cultures did not appear to hold older adults in higher esteem than Western ones [25]. The authors also found that recent rises in population ageing significantly predicted negative elder attitudes, after adjusting for industrialization. However, to the best of our knowledge, no publication has investigated specific cultural differences in feelings of usefulness among older adults in Europe. In this regard, our study provides novel data regarding the combinations of factors that may be associated with negative self-appraisal in older age and low self-perceived usefulness. Programmes to promote ageing well must take account not only of individual needs, but also of the environment and the cultural and social structures within which older persons construct their lives [26].

The strengths of our study include the fact that, to the best of our knowledge, it is the first study to have investigated the feeling of utility among older individuals in Europe. Our results give a snapshot of older Europeans’ feeling of usefulness. We also showed that among these older persons, there is a small proportion who are particularly vulnerable. The challenge here is to use appropriate approaches to reinforce social links in this group in order to enhance their feeling of utility and give them a better sense of ageing well (e.g. Class 2 and 3). The results of any future interventions to this end could use our findings as a benchmark against which to measure their efficacy.

Our study also has some limitations that deserve to be mentioned. The statistical representativeness of our study may not be optimal, as only four European countries were included. However, the use of the quota method made it possible to obtain a sample structure similar to that of the European population. It is also possible that our study sample underestimates the number of very lonely older persons, unfamiliar with digital technologies and particularly socially vulnerable. Indeed, the Ipsos ® panel was used for this study and is addressed to subjects who have an internet connection in their home, are able to use it, and who have a certain inclination to respond to surveys. Therefore, respondents may have been among the least lonely of their age group [27, 28], with preserved cognitive function [29] and better well-being [30]. Finally, extrapolation of our data may not be possible to countries and cultures outside of Europe, where healthcare and retirement systems may be considerably different.

## Conclusion and implications

This study provides new insights into the perception that older European subjects have of their own usefulness. Although the majority of respondents had a feeling of utility, there are two classes of the population who have a lesser sentiment of being useful. This poor perception of their own usefulness is also associated with more loneliness and reduced quality of life.

Our findings suggest that a non-negligible proportion of older subjects should be targeted for specific interventions aimed at enhancing their feeling of utility, to improve and support their feeling of successful ageing.

## Supplementary Information

Below is the link to the electronic supplementary material.

Supplementary file1 (DOCX 55 KB)

## Data Availability

Not applicable.
