# Supplementary material for: Being useful among persons aged over 65: social representations from a cross-sectional European study
Source: Aging Clin Exp Res. 2021 Jan 3;33(9):2565–72. doi: 10.1007/s40520-020-01767-x (PMC8429162; doi:10.1007/s40520-020-01767-x)
Supplement: Supplementary file 1 — Supplementary file1 (DOCX 55 KB) [file 40520_2020_1767_MOESM1_ESM.docx]

Part 1: General information

Question 1: Gender

1. Male
2. Female

Question 2: Country

1. France
2. Belgium
3. Italy
4. Germany

Question 3: Age (in years) _____

Question 4: Region (depending on the chosen country in Q2)

1. France: Ile-de-France/Northwest/Northeast/Southwest/SoutheastEst
2. Belgium: Center (Brussels)/North (Flanders)/South (Wallonia)
3. Italy: Northwest /Northeast/Centre/South/Islands
4. Germany (Nielsen areas): I/II/IIIa/IIIb/IV/V/VI/VII

Question 5: Market size (depending on the chosen country on Q2)

1. France: rural/ 2000-19999 inhabitants/20000–99999 inhabitants/≥100000 inhabitants/Paris
2. Belgium-Italy-Germany: <100 inhabitants/km^2^//100-499 inhabitants/km^2^//≥500 inhabitants/km^2^

Question 6 : Are you the head of your household ?

1. Yes
2. No

Question 7: Occupation of the head of the household

1. Manager or high-level professional
2. Technicians, clerks, service workers
3. Labou**rers, elementary occupations, armed forces
4. Unemployed, inactive

Question 8: Occupation of the respondent (only if the respondent is not the head of the household)

1. Manager or high-level professional
2. Technicians, clerks, service workers
3. Labourers, elementary occupations, armed forces
4. Unemployed, inactive

Question 9: Choose the answer that best describes you:

1. Currently working
2. Currently retired
3. You never worked

Question 10 : How many people currently comprise your household ? (count everyone, including yourself)

/_/_/ person(s) [authorize a numerical answer from 1 to 99]

Question 11 : Please indicate your marital status (choose only one answer):

| **French** | **Dutch** | Italian | German | *English translation* |
| --- | --- | --- | --- | --- |
| **Situation de famille** | **Burgerlijke staat** | **Stato civile** | **Familienstand** | ***Marital status*** |
| 1. Célibataire | 1. Vrijgezel | 1.Mai sposato (Single) | 1. Ledig (noch nie verheiratet) | *1. Single (never married)* |
| 2. En union libre | 2. In een relatie / samenwonend | 2.Convivente | 2. Eheähnliche Lebensgemeinschaft | *2. Living maritally* |
| 3. Marié(e) | 3. Getrouwd | 3.Sposato | 3. Verheiratet | *3. Married* |
| 4. Séparé(e) | 4. Feitelijk gescheiden | 4.Separato | 4. Getrennt lebend | *4. Separated* |
| 5. Divorcé(e) | 5. Uit de echt gescheiden | 5.Divorziato | 5. Geschieden | *5. Divorced* |
| 6. Veuf/ve | 6. Weduwnaar/weduwe | 6.Vedovo | 6. Verwitwet | *6. Widowed* |

Question 12 : How many children do you have ? (single answer only)

1. 0
2. 1
3. 2
4. 3
5. 4 or more

Question 13: How many grandchildren do you have? (single answer only)

1. 0
2. 1
3. 2
4. 3
5. 4 or more

Question 14: Are you a regular caregiver (either the main caregiver, or with help from one or more persons in your entourage) for an elderly, sick or handicapped person (whether they live with you or not)? Single answer

1. Yes, I current am a caregiver
2. I was in the past, but I am no longer a caregiver
3. No, I have never been a caregiver

Question 15 : Select the highest level of education you attained. Single answer

1. Low : Primary and/or some secondary school
2. Medium: Secondary school and/or 1 to 2 years of higher education
3. High: University degree or higher

Question 16 : What is the net monthly income of your household, after tax ?

| 1. 0 € –499 € |
| --- |
| 1. 500 €-– 999 € |
| 1. 1000 € –1199 € |
| 1. 1200 € –1399 € |
| 1. 1400 €– 1699 € |
| 1. 1700 € –1999 € |
| 1. 2000 € – 2299 € |
| 1. 2300 €–2999 € |
| 1. 3000 € –4499 € |
| 1. 4500 €–5999 € |
| 1. 6000 € and over |
| 1. I prefer not to answer |

*Note: for analysis, these categories were collapsed into low (0 to 1699 €), medium (1700 to 2999 €) and high (≥3000 €).*

Part 2 : Your sense of usefulness

Question 17 : Which statement best describes your current situation (single answer only)

1. You manage to save a lot of money
2. You manage to save some money
3. Your income is just about enough to meet your needs
4. You live partially off your savings
5. You are in debt, and have one or more credits or loans from your entourage

Question 18 : In your opinion, which situation best describes the majority of older people (single answer only)

1. They manage to save a lot of money
2. They manage to save some money
3. Their income is just about enough to meet their needs
4. They live partially off their savings
5. They are in debt, and have one or more credits or loans from their entourage

Question 19 : How well would you say you accept your age ? (single answer only )

1. Very well
2. Quite well
3. Quite badly
4. Very badly

Question 20 : Would you say that most older people accept their age well ? (single answer only)

1. Very well
2. Quite well
3. Quite badly
4. Very badly

Question 21 : To what extent do you agree with each of the following statements? (Single answer per row, random between rows)

*In column:*

1. Strongly agree
2. Agree
3. Disagree slightly
4. Strongly disagree

*In rows, randomize*

1. I have confidence in the future
2. I feel physically and psychologically fulfilled
3. I like to enjoy myself as much as ever
4. I sometimes feel younger than my children’s generation
5. I take life as it comes and avoid thinking about tomorrow
6. I don’t feel that I have changed much in the past 10 years
7. I like novelty, and things that are new to me.
8. I do my best to prepare for old age

Question 22: How often do you experience the following situations? (Single answer per row)

*In column:*

1. Very often
2. Quite often
3. Not very often
4. Never

*In rows, randomize*

1. Romantic feelings
2. Feel physically attracted to someone
3. Have sexual intercourse
4. Seduce someone
5. Go dancing, or to a party
6. Feel much younger than your age
7. Have the desire to start life over

Question 23: In your opinion, how often do most older people experience the following situations? (Single answer per row)

*In column:*

1. Very often
2. Quite often
3. Not very often
4. Never

*In rows, randomize*

1. Romantic feelings
2. Feel physically attracted to someone
3. Have sexual intercourse
4. Seduce someone
5. Go dancing, or to a party
6. Feel much younger than your age
7. Have the desire to start life over

Question 24 : Currently, when thinking about your place in society, would you say….? (single answer)

1. That you are a citizen in your own right, treated rather better than others
2. That you are a citizen in your own right, like everyone else
3. That you are not a citizen in your own right, but rather you are treated less well than others

Question 25 : When thinking about the place of older people in society, would you say….? (single answer)

1. That they are citizens in their own right, treated rather better than others
2. That they are citizens in their own right, like everyone else
3. That they are not citizens in their own right, but rather they are treated less well than others

Question 26 : Do you use internet to do the following ? (Single answer per row, random between rows)

*In column*

1. Every day, or almost every day
2. At least once a week
3. Two or three times per month
4. Once a month
5. Less often than once a month
6. Never

*In rows, randomize*

1. Send emails
2. Use Skype or equivalent application to chat with my family
3. Buy things online (items, tickets, shopping, etc)
4. Search for information that interests me, participate in forum discussions
5. Visit online dating sites
6. Manage my finances
7. Visit websites for health information
8. Listen to music, watch films
9. Use social networks (Facebook, Twitter…)

Question 27 : Currently, do you do the following things ? (Single answer per row)

*In column:*

1. Frequently
2. Quite often
3. Sometimes
4. Rarely
5. Never

*In rows, randomize*

1. Mind your grandchildren
2. Provide home-based academic support/tuition
3. Financially support your children or other members of your family
4. Take part in demonstrations, sign petitions
5. Have family members to visit for holidays
6. Participate in an association / a community / a support group
7. Help a dependent older person in your family or entourage (neighbour, friend, etc.)
8. Give advice to your children or grandchildren (about career, school, relationships, etc.)
9. Help people who do not live in your household to perform certain household tasks (repairs, cooking, cleaning, etc.
10. Accompany your grandchildren on school outings
11. Take arts and crafts classes (painting, drawing, DIY, etc.)
12. Organize / initiate family gatherings
13. Continue activities in relation with your previous profession / pursue previous activities
14. Discover new things, keep abreast of innovation

Question 28 : Generally, in everyday life, do you ever feel lonely during the day? (Single answer)

1. Every day or almost every day
2. Most days
3. At least every other day
4. Only some days
5. Rarely or never

Question 29 : How often do you think that most older people feel lonely? (Single answer)

1. Every day or almost every day
2. Most days
3. At least every other day
4. Only some days
5. Rarely or never

Question 30 : Do you suffer from these feelings of loneliness? (Single answer)

1. Yes, very much
2. Yes, somewhat
3. No, not really
4. No, not at all

Question 31 : When you are feeling down or poorly, can you count on the following people to provide help or support? (Single answer per row)

*In column:*

1. Yes, very much
2. Yes, somewhat
3. No, not really
4. No, not at all
5. Not applicable

*In rows, randomize:*

1. Your children
2. Your grandchildren
3. Your neighbours
4. Your spouse
5. Your friends
6. Your home-help (the person who does your cleaning/ prepares your meals / shopping, etc.)
7. Your parents
8. Members of a local association that you are a member of, or with which you are in contact

Question 32 : When older people you know are feeling down or poorly, in general, do you think they can count on the following people to provide help or support? (Single answer per row)

*In column:*

1. Yes, very much
2. Yes, somewhat
3. No, not really
4. No, not at all

*In row, randomize :*

1. Their children
2. Their grandchildren
3. Their neighbours
4. Their spouse
5. Their friends
6. Their home-help (the person who does your cleaning/ prepares your meals / shopping, etc.)
7. Their parents (if still alive)
8. Members of a local association that they are members of, or with which they are in contact

Question 33 : Currently, would you say that life is a source of pleasure for you? (Single answer)

1. Yes, very much
2. Yes, somewhat
3. No, not really
4. No, not at all

Question 34 : In general, do you think that life today is a source of pleasure for most older people? (Single answer)

1. Yes, very much
2. Yes, somewhat
3. No, not really
4. No, not at all

Question 35 : Which statement best describes your current situation … (Single answer)

1. You are in full possession of your faculties, you can do everything alone: get abaout, organise your daily life (meals, hygiene, housework, shopping…)
2. You have some minor health problems or minor difficulties and you need a bit of help with some tasks: e.g. for getting about or for organizing your daily life (meals, hygiene, housework, shopping…)
3. You have major health problems or major difficulties and you need substantial help to manage certain tasks: e.g. for getting about or for organizing your daily life (meals, hygiene, housework, shopping…)

Question 36 : At what age does a person start to be old ? (Numeric answer – allow 18 to 100) ____

Question 37 : For the good of society as a whole, would you be in favour of, or against the systematic implementation of the following measures when people get to a certain age: (Single answer per row)

*In column:*

1. Strongly in favour
2. In favour
3. Against
4. Strongly against

*In rows, randomize:*

1. Limit their right to vote, particularly on subjects that concern the future of young generations
2. Prevent them for holding positions of responsibility (political, in business, etc.)
3. Prevent them from holding positions of responsibility within associations
4. Oblige them to stop working once they have reached retirement age
5. Limit their driving rights, including by making them do a driving test (with the possibility that their licence could be withdrawn)

Question 38 : Personally, would you be shocked or not if an older person in your entourage did the following? (Single answer per row)

*In column:*

1. I would be shocked
2. I would not be shocked, but I would find it unusual
3. I would not be shocked, and I would not find it unusual

*In rows, randomize:*

1. If someone refused to retire, and continued to work
2. If someone got involved in politics and ran for election
3. If someone took up studies with a view to beginning a new activity
4. If someone started a new life, remarried
5. If someone decided to have a baby
6. If someone decided to start a business
7. If someone made risky financial investments

Question 39 : To be happy in life, apart from good health, what are the most important things in your life? Please indicate the three most important things— rank them in order of importance from one to three (Single answer per column and per row)

*In column:*

1. First
2. Second
3. Third

*In rows, randomize:*

1. Helping, advising others
2. Understanding the times we live in
3. Having leisure activities (travel, sport, DIY, etc.)
4. Being involved in an association or have a professional activity (including part-time)
5. Having friends
6. Having children
7. Having grandchildren
8. Having money
9. Having religious faith
10. Being in love
11. Being able to get around alone
12. Having sexual intercourse
13. Sharing, passing on things
14. Taking care of oneself
15. Stay living at home

Question 40 : To be happy in life, apart from good health, what do you think are the most important things in life for most older people ? Please indicate the three most important things— rank them in order of importance from one to three (Single answer per column and per row)

*In column:*

1. First
2. Second
3. Third

In row, randomize:

1. Helping, advising others
2. Understanding the times we live in
3. Having leisure activities (travel, sport, DIY, etc.)
4. Being involved in an association or have a professional activity (including part-time)
5. Having friends
6. Having children
7. Having grandchildren
8. Having money
9. Having religious faith
10. Being in love
11. Being able to get around alone
12. Having sexual intercourse
13. Sharing, passing on things
14. Taking care of oneself
15. Stay living at home

Question 41 : Overall, currently, do you feel …… (single answer per row)

*In column*

1. Yes, very much
2. Yes, somewhat
3. No, not really
4. No, not at all

*In rows, rotate items :*

1. Useful
2. Satisfied with your life

Question 42 : In general, do you think that older people feel…. (single answer per row)

*In column*

1. Yes, very much
2. Yes, somewhat
3. No, not really
4. No, not at all

*In row, rotate items:*

1. Useful
2. Satisfied with their life

Question 43 : In general, do you think it is still possible to help others in the following situations (single answer per row):

*In column*

1. Yes, very much
2. Yes, somewhat
3. No, not really
4. No, not at all

*In row, randomize:*

1. When you are retired
2. When you have lost your autonomy and you cannot go outside of your home
3. When you have lost your autonomy and you start to lose your memory
4. When you are living in a nursing home

Question 44 : For each of the following activities, please grade it according to the feeling of utility you get from it. A grade of 0 means that you don’t feel useful at all doing that activity, and 10 means that you get a strong feeling of utility from doing that activity. You can use all values between 0 and 10. Please give one grade per row (Display scale 0 to 10 (same layout as in the satisfaction questions at the end of the survey)

1. I__I__I /10
2. Not applicable

*In row, randomize:*

- 1. Participate in housework (set the table, clear away the table, clean up, dust, make your bed….)
  2. When someone asks your advice or your opinion
  3. Call your family when they are unwell and need someone to lift their spirits
  4. Go shopping in local shops and contribute to the local economy (hairdresser, bakery, etc.)
  5. Cook for others
  6. Cook for yourself
  7. Help a child to do homework
  8. Take care of a pet
  9. When someone asks you to tell stories of things your remember, or the history of your family
  10. Take part in group leisure activities
  11. Do paperwork (administrative, bank/finance, taxes)
  12. Keep certain traditions alive (organise a meal for a religious feast, incite your family to attend a ceremony, etc.)
  13. Help your family financially
  14. Make a donation to an association
  15. Pass on knowledge (recipes, DIY techniques, gardening, etc.)
  16. Do favours for your family (mind grandchildren, do shopping or ironing for someone…)
  17. Do volunteer work in an association
  18. Get involved in community life in your town or your area
  19. Go on the internet regularly (to read the news, do administrative tasks, send/receive messages to and from family, etc.)
  20. Manage your daily affairs without needing to ask your family for help

Question 45 : For each of the following activities, please grade it according to the feeling of utility older people get from it, in your opinion. A grade of 0 means that they do not feel useful at all doing that activity, and 10 means that they get a strong feeling of utility from doing that activity. You can use all values between 0 and 10. Please give one grade per row.

1. I__I__I /10
2. Not applicable

*In row, randomize:*

1. Participate in housework (set the table, clear away the table, clean up, dust, make your bed….)
2. When someone asks their advice or their opinion
3. Call their family when one of their family is unwell and needs someone to lift their spirits
4. Go shopping in local shops and contribute to the local economy (hairdresser, bakery etc)
5. Cook for others
6. Cook for themselves
7. Help a child to do homework
8. Take care of a pet
9. When someone asks them to tell stories of things they remember, or the history of their family
10. Take part in group leisure activities
11. Do paperwork (administrative, bank/finance, taxes)
12. Keep certain traditions alive (organise a meal for a religious feast, incite their family to attend a ceremony etc)
13. Help their family financially
14. Make a donation to an association
15. Pass on knowledge (recipes, DIY techniques, gardening, etc.)
16. Do favours for their family (mind grandchildren, do shopping or ironing for someone…)
17. Do volunteer work in an association
18. Get involved in community life in their town or their area
19. Go on the internet regularly (to read the news, do administrative tasks, send/receive messages to and from family, etc.)
20. Manage their daily affairs without needing to ask their family for help

Question 46 : Are there any older persons in your entourage who do the following: single answer per row.

*In column*

1. Yes, regularly
2. Yes, sometimes
3. No, rarely or never
4. Not applicable

*In rows, randomize:*

1. Help you out financially
2. Advise / help you to find solutions when you have a problem
3. Help you to perform domestic tasks (shopping, ironing, housework, gardening, DIY, etc.)
4. Help you with your children (collect them from school, help them to do their homework, mind them….)
5. Provide you with moral support when you feel down
6. Support you when you are sick
7. Pass on information / knowledge (e.g. cooking recipes, family stories, important events they experienced….)
8. Act as an intermediary when there are problems between you and other members of the family
9. Remind you of what is truly important (values that are dear to them: e.g. religion, unconditional love of the family, etc.)

Question 47 : Do you do the following for (or with) older persons in your entourage ? Single answer per row.

*In column*

1. Yes, regularly
2. Yes, sometimes
3. No, rarely or never
4. Not applicable

*In rows, randomize:*

1. Help them out financially
2. Advise / help them to find solutions when they have a problem
3. Help them to perform domestic tasks (shopping, ironing, housework, gardening, DIY, etc.)
4. Help them because they are dependent or suffer from a serious and/or chronic illness
5. Provide them with moral support when they feel down
6. Support them when they are sick
7. Les soutenir quand elles sont malades
8. Pass on information / knowledge (e.g.  computer skills, news, cooking recipes administrative help...)
9. Act as an intermediary when there are problems between them and other members of the family
10. Remind them of what is truly important (values that are dear to you, e.g. religion, unconditional love of the family, etc.)

Question 48 : Do the following people give you the impression that you are important to them? Please indicate one response per row.

*In column*

1. Yes, very much
2. Yes, somewhat
3. No, not really
4. No, not at all
5. Not applicable

*In rows, randomize:*

1. Your children
2. Your grandchildren
3. Your neighbours
4. Your friernds
5. Your parents/ elders
6. Your pet
7. Medical professionals that you are in contact with (doctor, nurse…)
8. Society as a whole
9. Public authorities
10. Associations and volunteers around where you live

Question 49 : When younger people from your family help you out, what do you think their main motives are ? Please indicate two answers, and rank them first and second.

*In column*

1. First
2. Second

*In rows, randomize:*

1. A sense of duty and responsibility
2. The affection they have for me
3. It makes them feel useful
4. It’s only normal to do so
5. They are grateful to you for the support you have given them
6. You help them financially in return

Question 50 : When you help older people, what are your main motives? Please indicate two answers, and rank them first and second. Single answer per row and per column.

*In column*

1. First
2. Second

*In row, randomize:*

1. A sense of duty and responsibility
2. The affection I have for then
3. It makes me feel useful
4. It is only normal to do so
5. I am grateful to them for the support they have given me
6. They help me financially in return

Question 51 : In the past 5 years, has anyone in your family moved to a nursing home ? Single answer.

1. Yes, one or more persons
2. Nobody

Question 52 (if you answered “Nobody” to question 51, go to question 56) : Do you (or did you) visit them? Single answer

1. Often
2. Sometimes
3. Rarely
4. Never

Question 53 (if you answered “Nobody” to question 51, go to question 56): In your opinion, do you visit….. Single answer, randomize

1. Often enough
2. Not often enough

Question 54 (if you answered “Often” or “Sometimes” to question 52, go to question 55): Among the following, which reasons best explain why you do not go (did not go) to visit more often? Please choose three answers and rank them as 1st, 2^nd^ and 3rd. Single answer per row and per column

*In column*

1. First
2. Second
3. Third

*In rows, randomize:*

1. There is/was no point because the person is not/was not in full possession of their faculties
2. It is/was too far from my home
3. The person does not/did not want to have any visitors
4. The person is not/was not close to me
5. Other people already go/went to see the person often
6. I don’t like going to nursing homes, they are a sad kind of place.
7. The person does not/did not want to see me.

Question 55 (if you answered “Rarely” or “Never” to question 52, go to question 56) : When you went to visit, do you (or did you) do the following things with the person (please choose one item per row):

*Single answer per row*

*In column*

1. Yes, because they need/needed to do it, or want/wanted to do it
2. No, because they do no’t/did not need to do it, or do not/did not want to do it.

*In rows, randomize:*

1. Ask them for advice
2. Give them advice
3. As them to tell you about things in their life (show you photos, etc…)
4. Tell them about things from your life (show them photos, etc…)
5. Ask them to show you around the nursing home, and show you their room
6. Let you take them out to dinner in a restaurant
7. Ask then to introduce you to the persons they get/got on best with
8. Watch television or listen to music with them
9. Read them a book, newspaper or letters
10. Ask them if there is/was anything wrong in the nursing home
11. Call them on the phone when you cannot/could not go to visit
12. Talk to them using applications such as Facebook, Instagram, skype, etc.
13. Participate with them in activities proposed in the nursing home
14. Participate with them in individual activities, other than those proposed by the nursing home
15. Participate with them in the running of the nursing home (social committee, menu committee….)
16. Go to appointments with them or go on outings outside of the nursing home.

Question 56 : How important do you think it is for older people living in a nursing home to be able to do the following things, if they want to (and with help, if necessary), so that they can feel more useful: Please choose one response per row. Single answer per row:

*In column*

1. Essential
2. Important but not essential
3. Less important
4. To be avoided

*In rows, randomize:*

1. Make their bed and tidy their personal belongings
2. Prepare, or participate in the preparation of their own meals
3. Have a pet
4. Do their own cleaning, or part of the cleaning in their room
5. Wash themselves whenever they want to during the day
6. Participate in the daily life of the nursing home (set the table, etc.)
7. Get involved in the committees and associations within the nursing home
8. Help others in their family life (when they are feeling down, or have problems)
9. Participate in debates/ discussions
10. Have an internet connection
11. Choose what they want to wear themselves
12. Decide on their own breakfast and lunch times
13. Spend money, buy small items
14. Give tips if they so desire (e.g. to the hairdresser, etc.)
15. Do their own laundry
16. Attend activities outside of the nursing home
17. Go out of the nursing home whenever they want to
18. Have family members to visit

Question 57 : Do you think it is possible to live in a nursing home and feel …

Single answer per row

*In column*

1. Yes, very much
2. Yes, somewhat
3. No, not really
4. No, not at all

*In row, rotate items:*

1. Useful
2. Happy with life
